# Arkansas Hot Springs

**Published:** 1877-07

**Authors:** 


THE editor of the *St. Louis Clinical Record*, speaks from personal observation of the Arkansas Hot Springs as follows: "We believe them greatly over-rated. That the treatment of syphilis, gout, and rheumatism, in their chronic forms, is remarkably successful at these springs, is a fact too well attested to admit of dispute. But that the waters in themselves possess any remarkable virtues seems to us altogether another matter. Enormous doses of mercury and iodide of potassium are given with a fearlessness—we might say recklessness—that is somewhat astonishing to one who has given no attention to the other treatment enjoined. Immense quantities of water at a high temperature are drunk, and with this frequent hot baths are given. These powerful alteratives are thus hurried through the system and rapidly eliminated. The process of repair and change of structure are thus greatly hastened. If the kidneys and skin are in fair working order, and the stomach does not get irritated, the improvement is, of necessity, rapid.

"Four, five, six, seven, and even eight hundred grains of the iodide have been given in the twenty-four hours, so we are informed. The stomach must first be guarded by demulcent drinks, of course.

"The bin-iodide and corrosive chloride of mercury, inunctions with the oleate of mercury, and calomel vapor baths, are freely employed. We saw no case—heard of no case of the cure of syphilis by the baths and drinking of the waters alone. A few cases—one or two—of cure of chronic rheumatism by the waters alone, without medicine, were reported to have occurred.

"The claim set up and defended so vigorously by interested parties and by deluded patients, of the marvelous, even supernatural virtues of these springs in the cure of chronic diseases, is absurd in the extreme, when all the facts are considered.

"If a physician is able to secure a perfect following out of his rules by his patient; can prevail upon him to drink the proper amount of hot pure water, and take as many hot water and vapor baths at home, and will have the courage to give as much mercury and iodide of potassium at home, he can cure him as rapidly and effectually at home as at the Hot Springs.

"There is one thing that the honorable, conscientious physician cannot do, however; he cannot impose upon the credulity of his patient, and gain the aid of those powerful co-operative agents, wonder and expectation, which the Hot Springs "resident physician" can do. These are auxiliaries too often despised, too often employed by the quack, to the benefit of the patient and the discomfort of the honorable practitioner.

"We give some of our Hot Springs confreres credit for being themselves the dupes of their own credulity in the matter of faith in the wonderful virtues of the waters. We cannot believe that they are all dishonest. At the same time we cannot believe that their faith is well founded. The weakness of their trust is fully demonstrated by the reliance they place upon the most potent agencies known to science, and their unwillingness to trust *any case* to the healing influence of the waters alone."—*Ohio Medical Recorder.*

